# Supplementary material for: Interprofessional perceptions of emotional, social, and ethical effects of multidrug-resistant organisms: A qualitative study
Source: PLoS One. 2021 Feb 22;16(2):e0246820. doi: 10.1371/journal.pone.0246820 (PMC7899372; doi:10.1371/journal.pone.0246820)
Supplement: S1 File — (DOCX) [file pone.0246820.s002.docx]

**
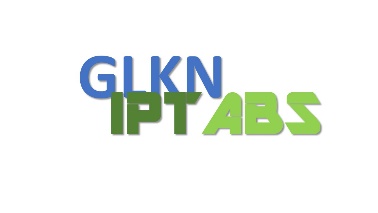
AUFKLÄRUNGS UND INFORMATIONSBOGEN**

IPTABS PROJEKT

Sehr geehrte Kursteilnehmer, liebe Kollegen

Mit dem IPTABS-Projekt möchten wir Sie nicht nur im Antibiotic Stewardship schulen, sondern nutzten die Methode der FMEA als Lehrmethode sowie eine Fokusgruppe, um die Verbindung von Emotionen und Multiresistenten Erregern genaue zu erforschen.

Die Ergebnisse dieser beiden Methoden sind auf Ihre Person bezogen anonym. Um die anonymen Ergebnisse statistisch auswerten, zur Hypothesengenerierung für weitere Projekte sowie für die Ausbildungsforschung mit Publikation der Ergebnisse in einer medizinischen Fachzeitschrift nutzen zu dürfen, bedarf es Ihres mündlichen Einverständnisses (ein schriftliches würde die Anonymität aufheben).

Wenn Sie der Nutzung der anonymisierten Ergebnisse zur Lehr- und Lernforschung NICHT zustimmten, wenden Sie sich bitte an den Kursleiter

Für Fragen zum Studienkonzept, zur Datensicherheit und der Datenanalyse stehe wir Ihnen jederzeit zur Verfügung.

Bei Fragen zum Studienprojekt wenden Sie sich bitte an

Oberarzt Dr.med. Stefan Bushuven DESA EDIC

Institut für Anästhesiologie, Intensivmedizin, Notfallmedizin und Schmerztherapie

Virchowstrasse 10

78224 Singen

Tel.: 07731 89- 2000

Email: [stefan.bushuven@glkn.de](mailto:stefan.bushuven@glkn.de)

**
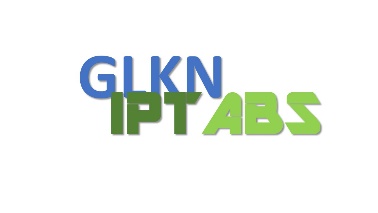
EINVERSTÄNDNISERKLÄRUNG**

Fotodokumentation- IPTABS PROJEKT

Sehr geehrte Kursteilnehmer, liebe Kollegen

Wir bitten Sie um Ihre Erlaubnis während des Kurses Fotos sowie ggf. Videoaufnahmen von Ihnen in der Kursdurchführung anfertigen und für weitere Kurse sowie für interne Infoflyer, Präsentationen und Publikationen in medizinischen Fachzeitschriften nutzen zu dürfen.

Das Material wird nicht an Dritte Personen weitergegeben verwendet.

- ich wurde durch ___________________________ ausreichend auf die Verwendung der erhobenen Materialien **(gem. Datenschutzgesetz §4a)** aufgeklärt
- ich stimme der Verwendung von Fotos und Videoaufnahmen (z.B. Infoflyer, Präsentationen) sowie zur Publikation in medizinischen Fachzeitschriften zu
- ich wurde darüber informiert, dass ich meine Einwilligung jederzeit, auch ohne Nennung von Gründen zurückziehen kann

_____________________________________________

Datum, Ort, Unterschrift

Wir danken Ihnen für Ihre Bereitschaft zur Teilnahme

Bei Fragen zum Studienprojekt wenden Sie sich bitte an

Oberarzt Dr.med. Stefan Bushuven DESA EDIC

Institut für Anästhesiologie, Intensivmedizin, Notfallmedizin und Schmerztherapie

Virchowstrasse 10

78224 Singen

Tel.: 07731 89- 2000

Email: [stefan.bushuven@glkn.de](mailto:stefan.bushuven@glkn.de)
