# Supplementary material for: Interprofessional perceptions of emotional, social, and ethical effects of multidrug-resistant organisms: A qualitative study
Source: PLoS One. 2021 Feb 22;16(2):e0246820. doi: 10.1371/journal.pone.0246820 (PMC7899372; doi:10.1371/journal.pone.0246820)
Supplement: Attachment — Submitted filename: MRDO Manuscript Review.docx [file pone.0246820.s003.docx]

**Manuscript Review**

**Reviewer’s Information**

| E-Mail: | [navelisharma98@gmail.com](mailto:navelisharma98@gmail.com) |
| --- | --- |
| First Name: | Naveli |
| Last Name: | Sharma |
| Country: | India |
| Specialization: | Applied Psychology |

**Manuscript Information**

| Journal Name: | PLOS ONE |
| --- | --- |
| Manuscript Number: | PONE-D-20-25927 |
| Manuscript Title: | Emotional, social and ethical effects of Multidrug-  Resistant Organisms: A Qualitative Study |
| Date Received from Journal: | October 24, 2020 |
| Date to Send Review Report: | November 13, 2020 |

**REVIEWER’S BLIND REVIEW**

Bushuven and co-authors investigated the psychological effects of Multidrug-resistant organisms (MDRO) among hospital staffs, patients and their families. A salient feature of this study is the illustration of ‘biopsychosocial model’ in a passive manner, although the model has no particular relationship with this study. The authors developed a program where focus group discussion and survey was done indicating that such a program can be effectively replicated.

The manuscript is rich in information; however, improvement is required in overall presentation of the paper. Further, it would be preferable that the authors re-write the Introduction and Methodology with appropriate syntax, punctuations and provide a structure to the mentioned sections. For Example, changing the word ‘und’ to ‘and’ in row 7 of the Introduction; deleting the word evaluated in row 11 of Introduction; writing full forms instead abbreviations like SB, AD and others in analysis, or concisely mentioning about the method of analysis used and the coding approaches utilized to take out codes and themes under the ‘Analysis’ sub-section.

**Additional Revisions**

1. Since this study is a follow-up study, the former study was not separately summarized and was mixed with the present paper, therefore, creating ambiguity whether the program developed in this study, was part of the previous paper or not. Basically, the technical details should be expanded or bifurcated so that the readers can understand what the researchers want to say.
2. In table 1, Inductive code of ‘Unempathy’ (8) should be replaced with ‘Lack of Empathy’ under Theme: ANGER.
3. In figure 3 and figure 4, it would be preferable that the x-axis is be labelled for better understanding.
4. The study was portrayed subjectively rather than objectively. Thus, lacking in generalization.
5. Also, the authors solely focused on isolation precaution rather than the management of MDRO at an individual and community level.
6. Furthermore, the authors are off to a promising beginning, however, this study requires covert observations in order to increase the validity of the paper.

Moreover, the paper can be refined and not be oversimplified. Nevertheless, what I liked about the study is that it is in-depth and the data was accessible and downloadable on spread sheet. Even the figures and tables were comprehensive and well-concocted. In short, this study has ‘diverse data with requisite of rectification’.

**Reviewer’s Confidential**

**Comments to Editor**

The study appears to be sound, but the language of the study is cryptic at certain sections, making it difficult to follow. Finally, other than the aforementioned revisions, the study is pertinent to the field of psychology and medical sciences. It has a vast scope of improvement, but currently it does not meet the criteria of your esteemed journal.
